# Monotropein Improves Dexamethasone-Induced Muscle Atrophy via the AKT/mTOR/FOXO3a Signaling Pathways

**DOI:** 10.3390/nu14091859

**Published:** 2022-04-29

**Authors:** Piao Wang, Seok Yong Kang, Su Jin Kim, Yong-Ki Park, Hyo Won Jung

**Affiliations:** 1 Department of Herbology, College of Korean Medicine, Dongguk University, Gyeongju 38066, Korea; 18234071171@163.com (P.W.); yongki@dongguk.ac.kr (Y.-K.P.); 2 Korean Medicine R&D Center, Dongguk University, Gyeongju 38066, Korea; seokppo2@hanmail.net; 3 Department of Anesthesiology and Pain Medicine, College of Medicine, Dongguk University, Gyeongju 38066, Korea; sujink100@hanmail.net

**Keywords:** AKT/mTOR/FOXO3a signaling pathways, Atrogin1, muscle atrophy, monotropein, dexamethasone, MuRF1

## Abstract

The present study aimed to investigate the effects of monotropein (MON) on improving dexamethasone (DEX)-induced muscle atrophy in mice and C2C12 mouse skeletal muscle cells. The body weights, grip strengths, and muscle weights of mice were assessed. The histological change in the gastrocnemius tissues was also observed through H&E staining. The expression of myosin heavy chain (MyHC), muscle ring finger 1 (MuRF1), and muscle atrophy F-box (Atrogin1) and the phosphorylation of AKT, mTOR, and FOXO3a in the muscle tissues of mice and C2C12 myotubes were analyzed using Western blotting. MON improved muscle atrophy in mice and C2C12 myotubes by regulating catabolic states via the AKT/mTOR/FOXO3a signaling pathways, and enhanced muscle function by the increases of muscle mass and strength in mice. This suggests that MON could be used for the prevention and treatment of muscle atrophy in patients.

## 1. Introduction

Skeletal muscles are made up of proteins that provide energy to organs during the metabolic breakdown process and are associated with a variety of diseases, such as diabetes, cancer, and chronic kidney failure [1]. Individually, muscle atrophy increases the likelihood of adverse outcomes such as falls and fractures, impaired quality of life, and even death [2]. Recently, the proportion of elderly people in the population has been increasing and the incidence of muscle atrophy has been given more and more attention all around the world.

The pathogenesis of muscle atrophy is very complex and it has been speculated to be the result of reduced protein synthesis, enhanced proteolysis, and/or a mixture of both [3]. Recent evidence suggests that the proteolysis of the ubiquitin–proteasome system plays a key role in controlling muscle mass. In particular, it has been reported that ATP-dependent muscle-specific E3 ubiquitin ligases such as muscle ring finger 1 (MuRF1) and muscle atrophy F-box (Atrogin1) are highly expressed during muscle atrophy and go on to induce protein degradation in skeletal muscle tissue [4,5]. However, understanding the mechanisms that regulate muscle mass and function will provide therapeutic targets for the treatment of muscle atrophy.

*Morinda officinalis* (Morindae Radix) root is commonly used to enhance the strength of muscles and bones by nourishing the kidneys, as well as for treating rheumatism in the elderly. Monotropein (MON), one of the most abundant compounds of iridoid glycosides in Morindae Radix, is known to stimulate the effect of osteogenesis in vivo ovariectomy models by promoting angiogenesis and accelerating wound healing [6,7]. MON is also known to have various activities, such as the inhibition of pro-inflammatory cytokines [8,9], the induction of chondrocyte apoptosis and catabolic response in IL-1β-induced osteoarthritis mice [10], increasing bone density, the promotion of proliferation/differentiation in dexamethasone (DEX)-induced osteoblast injuries, and the prevention of nociception [11].

In a previous study, we found that Morindae Radix extract is effective in increasing muscle mass by stimulating muscle differentiation and mitochondrial biogenesis in type 2 diabetic mice and C2C12 cells [12]. To explore the possibility of drug development, we investigated the effects of MON, a main bioactive compound of Morindae Radix, on DEX-induced muscle atrophy in mice and C2C12 mouse muscle cells and identified the mechanism of action responsible for the anti-muscle atrophy effect of MON.

## 2. Materials and Methods

### 2.1. Cell Culture

The C2C12 cells, a mouse skeletal myoblast line (No. CRL-1772™, ATCC, Manassas, VA, USA), were maintained in Dulbecco Modified Eagle Medium (DMEM; Corning, New York, NY, USA) supplemented with 10% fetal bovine serum (FBS; Merck Millipore, Burlington, MA, USA) and 1% penicillin/streptomycin (Corning) at 37 °C in a 5% CO_2_ incubator. When a confluence of more than 90% was reached, the medium was changed to DMEM supplemented with 2% horse serum (HS; Thermo Fisher Scientific, Waltham, MA, USA) for 4 days to induce the differentiation of myoblasts into myotubes.

### 2.2. Treatment of Monotropein and Dexamethasone

After differentiation, the myotubes were divided into five groups as follows: the normal group (Nor), cells were cultured in DMEM supplement HS and DMSO which is a DEX vehicle solution; the dexamethasone-treated group (DEX), in which cells were treated with 100 μmol/mL of DEX (Cat. D4902, Sigma-Aldrich, St. Louis, MO, USA); and the monotropein-treated group (MON), in which cells were treated with DEX plus MON (25, 50 and 100 μmol/mL) (Cat. T6S1579, TargetMo, Wellesley Hills, MA, USA). DEX and MON were dissolved in DMSO and 1× PBS, respectively. All groups were harvested after the treatment of MON with or without DEX for 24 h, and then the next experiments were performed. The C2C12 cell treatment with MON was shown to cause no toxicity in the cell viability up to 300 μmol/mL (Figure S1).

### 2.3. Immunocytochemistry Staining

After the treatment of MON or DEX for 24 h in the C2C12 myotubes, the cells were fixed in 4% formaldehyde on ice for 20 min and permeabilized with 0.5 % Triton X-100 (Sigma-Aldrich, St. Louis, MO, USA). Subsequently, the cells were incubated with anti-MyHC antibodies (Cat. Sc-376157, Santa Cruz Biotechnology Inc., Dallas, TX, USA) at 4 °C overnight and an Alexa Fluor 488-conjugated anti-mouse antibody (Cat. A11001, Thermo Fisher Scientific, Waltham, USA). DAPI was also used for nucleus staining. The stained cells were observed under a fluorescence microscope (BioTek, Agilent CO., Santa Clara, CA, USA). The myotube diameter, length, and fusion index (the proportion of nuclei in myotubes to the total nuclei) were analyzed using Image J software (https://imagej.nih.gov/ij/, accessed on 1 February 2021).

### 2.4. Animals and Treatment

Eight-week-old male C57BL/6N mice were obtained from Koatech (Pyeongtaek, Korea). All animal studies were approved by the Use Committee of the Dongguk University (IACUC-2021-10) and performed in accordance with the guidelines for Institutional Animal Care. The mice were maintained under controlled conditions at 22 ± 3 °C and a 12 h/12 h light/dark cycle. They were given free access to food and water. After adaption for one week, all the mice were randomly divided into four groups (*n* = 8 animals per group) as follows: (I) the normal group was administered 0.9% saline, intraperitoneal injection of the same volume of PEG400 (Cat. 25322-68-3, Sigma-Aldrich, St. Louis, MO, USA) as DEX group subsequently (Nor group); (II) the dexamethasone was dissolved into PEG400 [13] and intraperitoneally injected DEX 10 mg/kg, then administrated 0.9% saline once a day for 10 days (DEX group); MON was dissolved into 0.9% saline. (III) One group was injected DEX and administered 40 mg/kg MON (MON40 group); and (IV) one group was injected DEX and administered 80 mg/kg MON (MON80 group). Two different concentrations of MON (40 and 80 mg/kg) were administered once a day. During treatment with drugs, the body weights of mice were measured once three days.

### 2.5. Measurement of Grip Strength

Briefly, mice were tested to determine their forelimb grip strength using a grip strength meter (Jeung Do Bio & Plant Co., Seoul, Korea) according to the instructions of the instrument on the 10th day. Mice were placed on the grid and held by their tail so that their limbs could grasp the grid. The mice were pulled backward by the tail gently until they released the grid. This test was performed three times per animal, and the average value was analyzed statistically.

### 2.6. Muscle Tissue Collection

At the end of experiment, mice were weighed and fasted for 24 h. After sacrifice, the gastrocnemius tissues on both hind legs were harvested and weighed, then stored at −80 °C for further testing with histopathological analysis and Western blotting.

### 2.7. Histology Observation

The gastrocnemius muscle tissues were fixed with 4% paraformaldehyde for 24 h then dehydration paraffin embedding, sliced into 4 μm sections, and stained with hematoxylin and eosin (H&E). The change in muscle tissue was observed under a microscope (original magnification = 200×). One hundred fibers were observed per animal, and the fiber cross-section area (μm^2^) was measured in the ImageJ software (https://imagej.nih.gov/ij/, accessed on 1 February 2021).

### 2.8. Western Blot Analysis

For the in vitro study, the C2C12 myotubes were added to the RIPA lysis and extraction buffer (Cat. 89901, Thermo Fisher Scientific), which included protease/phosphatase inhibitors, and then total proteins were isolated by centrifugation at 14,000 rpm for 20 min at 4 °C. For the in vivo study, gastrocnemius muscle tissues were homogenized using a homogenizer (T10 basic, IKA, Staufenim Breisgau, Germany) with tissue lysis buffer (Cat. 78510, Thermo Fisher Scientific) containing protease/phosphatase inhibitors, put on ice for 20 min, and centrifuged at 14,000 rpm at 4 °C for 20 min. The protein concentrations of the supernatants were measured using a protein assay solution. Equal amounts of each protein were separated into 8% acrylamide gels, and SDS-polyacrylamide gel electrophoresis (PAGE) was performed. After transferring the membranes and blocking with 5% skim milk, the membranes were incubated with the primary antibodies against MyHC (sc-376157, Santa Cruz, Dallas, CA, USA), MuRF1 (Cat. bs-2539R, Bioss Antibodies, Woburn, MA, USA), mTOR (Cat. 2972s, Cell Signaling, Danvers, MA, USA), p-mTOR (Cat. 5536s), AKT (Cat. 9272s), p-AKT (Cat. AF887, R&D), Atrogin1 (Cat. PA5-91959, Thermo Fisher Scientific), Myostatin (Cat. PA5-11936), p-FOXO3a (Cat. PA5-36816), and FOXO3a (Cat. PA5-27145) at 4 °C overnight. The membranes were then washed three times with 1× Tris-buffered saline (pH 7.4) containing 0.1% Tween-20 (TBST) buffer for 15 min. The membranes were incubated with the secondary antibodies, including anti-mouse IgG or anti-rabbit IgG (Bio-Rad, Hercules, CA, USA), for 2 h at room temperature and washed three times with 1× TBST buffer. The protein signals were measured under a ChemiDoc MP Imaging System (Bio-Rad) and the band density was quantified by densitometry using the ImageJ programming software (ImageJ, NIH, Bethesda, MD, USA). The expression of each target protein was normalized to β-actin (Sigma-Aldrich) as an internal control.

### 2.9. Statistical Analysis

All data are presented as means and standard deviations of the mean (mean ± SD; *n* = 8 per each group in vivo and *n* = 3 in vitro). A one-way analysis of variance (ANOVA) was used to analyze the statistical significance of the differences between the groups. The GraphPad Prism 5.0 (GraphPad Software, San Diego, CA, USA) software was applied to all statistical analyses. A *p*-value <0.05 was considered significant.

## 3. Results

### 3.1. Effect of MON on MyHC Expression in DEX-Treated C2C12 Myotubes

To assess the effect of MON on the improvement of muscle atrophy, we measured the expression of MyHC, as a marker of muscle maturation, in the C2C12 myotubes with DEX-induced muscle atrophy by immunocytochemistry and Western blot analyses.

**Figure 1:**
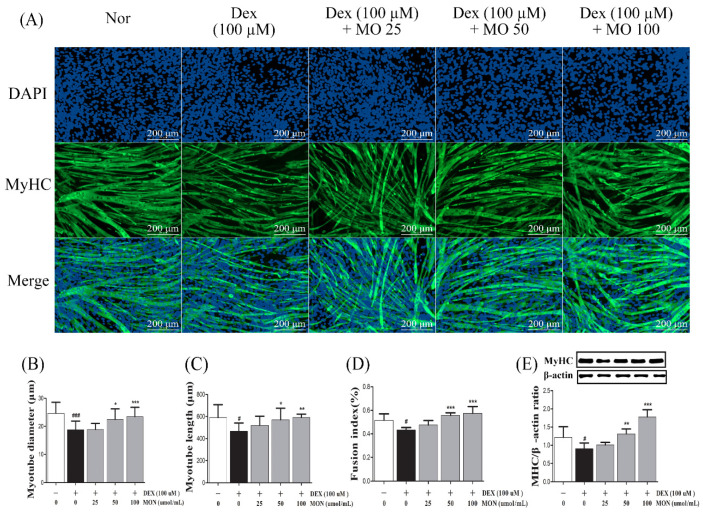
Effect of MON on MyHC expression in the DEX-stimulated C2C12 myotubes. Representative photographs of MyHC expression in C2C12 myotubes after immunocytochemical staining with anti-MyHC antibody (green) with DAPI (blue) under fluorescence microscopic observation (100×) (**A**). Myotube diameter (**B**), length (**C**), and fusion index (**D**) were measured. The expression of MyHC protein was detected in C2C12 myotubes by Western blotting. Relative expression of each protein was normalized to β-actin as an internal control (**E**). Each value is the mean ± SD of three independent experiments. # *p* < 0.05, ### *p* < 0.001 vs. Nor; * *p* < 0.05, ** *p* < 0.01, and *** *p* < 0.001 vs. DEX. Nor, normal group; DEX, dexamethasone-treated group; and MON, group-administered monotropein.

In the in vitro study, MyHC expression was observed in the myotubes by ICC staining (Figure 1A), and also measured the diameter (Figure 1B), length (Figure 1C), and fusion index (Figure 1D). As the result, DEX stimulation significantly reduced diameter (*p* < 0.001), length (*p* < 0.05), and fusion index (*p* < 0.05) compared with normal group. Treatment with MON significantly increased diameters (*p* < 0.05 for 50 μM and *p* < 0.001 for 100 μM) and lengths (*p* < 0.05 for 50 μM and *p* < 0.01 for 100 μM) in DEX-stimulated myotubes. In the 50 and 100 μM of MON treatment groups, the fusion index was also increased compared with DEX group (*p* < 0.001, Figure 1D). In addition, MON treatment at 50 μM (*p* < 0.01) and 100 μM (*p* < 0.001) significantly increased MyHC expression in DEX-stimulated myotubes (Figure 1E), which was consistent with ICC results.

### 3.2. Effect of MON on the Expression of Muscle Atrophy-Regulating Factors in DEX-Treated C2C12 Myotubes

To investigate the effect of MON on muscle atrophy, we measured the expression of muscle degradation enzymes (Atrogin1 and MuRF1) and a negative regulator of muscle growth (Myostatin) in the C2C12 myotubes with DEX-induced muscle atrophy by Western blotting.

**Figure 2:**
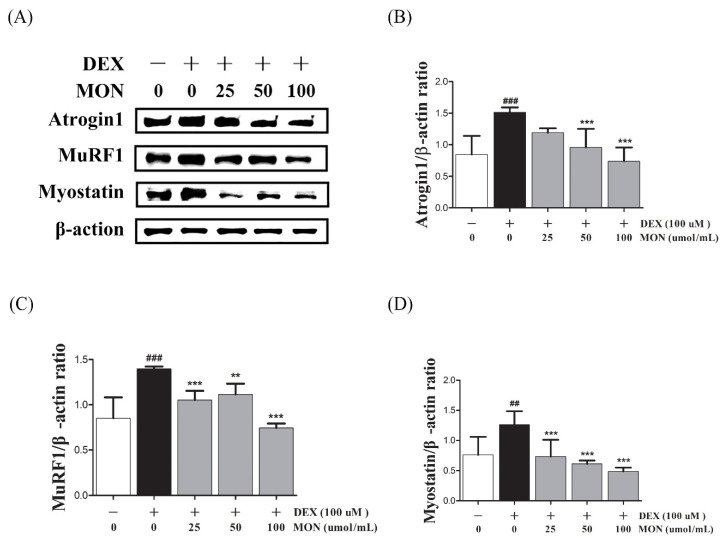
Effect of MON on the expression of Atrogin1, MuRF1, and Myostatin in DEX-stimulated C2C12 myotubes. The expression of Atrogin1, MuRF1, and Myostatin was determined in the C2C12 myotubes by Western blotting (**A**). Relative expression of each protein was normalized to β-actin (**B**–**D**). Each value is the mean ± SD of three independent experiments. ## *p* < 0.01, ### *p* < 0.001 vs. Nor; ** *p* < 0.01, *** *p* < 0.001 vs. DEX. Nor, normal group; DEX, dexamethasone-treated group; and MON, group-administered monotropein.

In the in vitro study, DEX stimulation was shown to significantly up-regulate the expression of Atrogin1, MuRF1, and Myostatin compared to the normal cells (Figure 2A). The treatment of MON at levels of 50 μM (*p* < 0.001) and 100 μM (*p* < 0.001) significantly decreased the expression of Atrogin1 in DEX-stimulated C2C12 myotubes (Figure 2B). The expression of MuRF1 (Figure 2C) and Myostatin (Figure 2D) was also significantly reduced after treatment with MON at levels of 25 μM (*p* < 0.001 for MuRF1 and *p* < 0.001 for Myostatin), 50 μM (*p* < 0.01 for MuRF1 and *p* < 0.001 for Myostatin), and 100 μM (*p* < 0.001 for MuRF1 and *p* < 0.001 for Myostatin) in C2C12 cells.

### 3.3. Effect of MON on the Expression of p-Akt, p-FoxO3a, and p-mTOR in C2C12 Myotubes

To identify the effect of the signaling pathway of MON on protein anabolism, we investigated the expression of mTOR, FOXO3a, and AKT as regulators of Atrogin1 and MuRF1 in the DEX-treated C2C12 myotubes by Western blotting.

**Figure 3:**
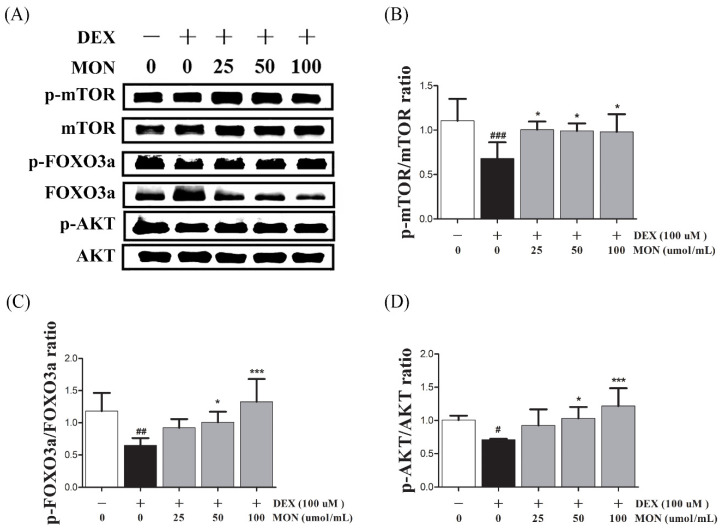
Effect of MON on the expression of p-Akt, p-FoxO3a, and p-mTOR in C2C12 myotubes. The phosphorylation of mTOR, FOXO3a, and AKT was determined by Western blot (**A**). MON increased expression of phosphorylation of each protein in C2C12 myotubes (**B**–**D**). Each value is the mean ± SD of three independent experiments. # *p* < 0.05, ## *p* < 0.01, and ### *p* < 0.001 vs. Nor; * *p* < 0.05, *** *p* < 0.001 vs. DEX. Nor, normal group; DEX, dexamethasone-treated group; and MON, monotropein-administrated group.

In the in vitro study, the phosphorylation of mTOR (*p* < 0.001), FOXO3a (*p* < 0.01), and AKT (*p* < 0.05) significantly decreased in DEX-stimulated C2C12 myotubes compared to in the normal cells (Figure 3). The phosphorylation of mTOR was significantly increased by treatment with MON at levels of 25 μM (*p* < 0.05), 50 μM (*p* < 0.05), and 100 μM (*p* < 0.05, Figure 3B). In addition, MON treatment at levels of 50 μM (*p* < 0.05 for FOXO3a and *p* < 0.05 for AKT, Figure 3C) and 100 μM (*p* < 0.001 for FOXO3a and *p* < 0.001 for AKT, Figure 3D) significantly increased the phosphorylation of FOXO3a and AKT, respectively.

### 3.4. MON Improved DEX-Treated Muscle Atrophy in Mice

**Figure 4:**
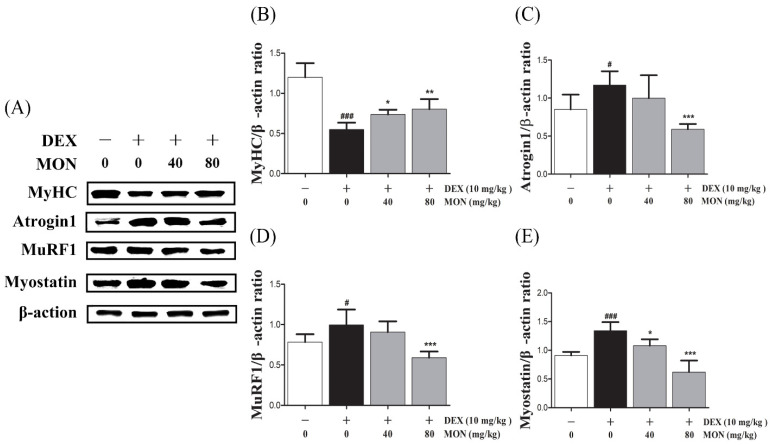
MON improved DEX-treated muscle atrophy in mice. The expression of MyHC, Atrogin1, MuRF1, and Myostatin was determined in gastrocnemius tissues of mice by Western blot (**A**). Relative expression of each protein was normalized to β-actin (**B**–**E**). Each value is the mean ± SD of three independent experiments. # *p* < 0.05, ### *p* < 0.001 vs. Nor; * *p* < 0.05, ** *p* < 0.01 and *** *p* < 0.001 vs. DEX. Nor, normal group; DEX, dexamethasone-treated group; and MON, monotropein-administrated group.

In the in vivo study, DEX stimulation was shown to significantly up-regulate the expression of Atrogin1, MuRF1, and Myostatin compared to the normal group (Figure 4A). The expression of MyHC in the gastrocnemius tissues of mice with DEX-induced muscle atrophy was significantly decreased (*p* < 0.001) compared with the normal group. The administration of MON to mice at levels of 40 mg/kg (*p* < 0.05) and 80 mg/kg (*p* < 0.01) significantly increased the expression of MyHC in their muscle tissues (Figure 4B). As expected, a significant decrease in the expression of Atrogin1 (*p* < 0.001, Figure 4C) and MuRF1 (*p* < 0.001, Figure 4D) was observed in gastrocnemius tissues by the administration of MON at a level of 80 mg/kg to mice with DEX-induced muscle atrophy. The expression of Myostatin was significantly decreased in atrophied muscle tissues (Figure 4E) after the administration of MON at levels of 40 mg/kg (*p* < 0.05) and 80 mg/kg (*p* < 0.001). These results indicate that MON prevents the induction of atrophy in muscle by the down-regulation of the negative regulators of muscle growth thus increasing MyHC expression.

### 3.5. Effect of MON on the AKT/mTOR/FOXO3a Signaling Pathway in DEX-Treated Mice

**Figure 5:**
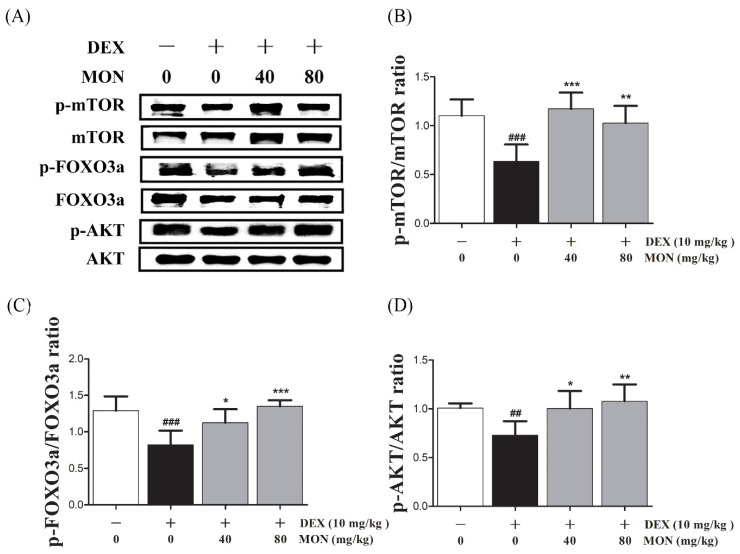
Effect of MON on the AKT/mTOR/FOXO3a signaling pathway in DEX-treated mice. The phosphorylation of mTOR, FOXO3a, and AKT was determined by Western blot (**A**). MON increased expression of phosphorylation of each protein in in gastrocnemius tissues of mice (**B**–**D**). Each value is the mean ± SD of three independent experiments. ## *p* < 0.01, ### *p* < 0.001 vs. Nor; * *p* < 0.05, ** *p* < 0.01 and *** *p* < 0.001 vs. DEX. Nor, normal group; DEX, dexamethasone-treated group; and MON, monotropein-administrated group.

Next, in the in vivo study, the phosphorylation of mTOR (*p* < 0.001), FOXO3a (*p* < 0.001), and AKT (*p* < 0.01) was significantly reduced in the gastrocnemius tissues of DEX-treated mice (Figure 5A). The phosphorylation of mTOR was significantly increased by MON administration at levels of 40 mg/kg (*p* < 0.001) and 80 mg/kg (*p* < 0.01, Figure 5B). In an analysis of the p-FOXO3a/FOXO3a ratio (*p* < 0.05 for 40 mg/kg and *p* < 0.001 for 80 mg/kg, Figure 5C) and p-AKT/AKT ratio (*p* < 0.05 for 40 mg/kg and *p* < 0.01 for 80 mg/kg, Figure 5D), a significant increase was also seen after MON administration. These results suggest that MON enhances protein anabolism by the regulation of the AKT/mTOR/FOXO3a signaling pathways in atrophied muscle.

### 3.6. Effect of MON on Structural Damage of Muscle Tissues in DEX-Treated Muscle Atrophy

To evaluate the beneficial effect of MON on the loss of muscle mass, we observed the structural changes in the gastrocnemius muscle tissues in mice with DEX-treated atrophy through H&E staining.

**Figure 6:**
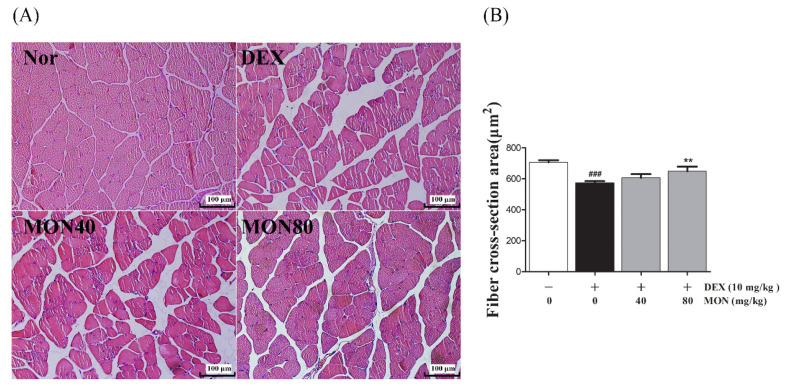
Effects of MON on the structural damage of muscle tissue in mice with DEX-treated muscle atrophy. The gastrocnemius tissues of mice were stained with H&E and observed under a microscope (200×) (**A**). The fiber cross-section areas of muscle tissues (μm^2^) were measured using Image J and expressed as mean ± S.D. (**B**). ### *p* < 0.001 vs. Nor; ** *p* < 0.01 vs. DEX. Nor, normal group; DEX, dexamethasone-treated group; and MON, group-administered monotropein at levels of 40 mg/kg or 80 mg/kg.

As shown in Figure 6, compared to the normal group, the myofibers of the DEX-treated control group showed morphological changes such as atrophy, loose arrangement, large interfibrous areas, and reduced numbers of myofibers. However, these symptoms were improved by the administration of MON to mice with DEX-treated atrophy (Figure 6A). The Fiber cross-section area was also significantly increased in mice with DEX-treated muscle atrophy after the administration of MON at levels of 80 mg/kg (*p* < 0.01) with a compact/regular arrangement (Figure 6B). These results indicate that MON prevents the structural damage of muscle tissues in mice with DEX-induced muscle atrophy.

### 3.7. Effect of MON on Muscle Mass and Function in DEX-Treated Muscle Atrophy

To evaluate the preventative effect of MON on muscle atrophy, we measured the muscle mass and grip strength of mice with DEX-treated muscle atrophy.

**Figure 7:**
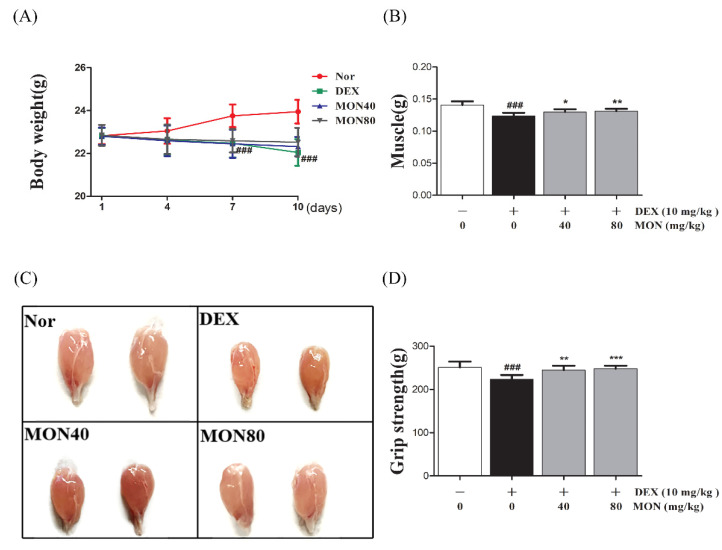
Treatment with MON improved muscle mass and strength in mice with DEX-induced muscle atrophy. The bodyweights (**A**) and gastrocnemius tissues (**B**,**C**) in each group of mice were measured. The grip strength (**D**) of the mice was tested. Each value is the mean ± SD. ### *p* < 0.001 vs. Nor; * *p* < 0.05, ** *p* < 0.01, and *** *p* < 0.001 vs. DEX. Nor, normal group; DEX, dexamethasone-treated group; and MON, group-administered monotropein at levels of 40 mg/kg or 80 mg/kg.

We found that mice treated with DEX showed significant (*p* < 0.001) decreases in their body weight (*p* < 0.001, Figure 7A) and weights of gastrocnemius tissues compared to the normal group (Figure 7B). The administration of MON at levels of 40 mg/kg (*p* < 0.05) and 80 mg/kg (*p* < 0.01) significantly increased the muscle weights of mice with atrophy (Figure 7B) compared to the DEX treatment group. In a grip strength test, a decrease in the grip strength of DEX-treated mice was also shown. However, MON administration at levels of 40 mg/kg (*p* < 0.01) and 80 mg/kg (*p* < 0.001) significantly improved the grip strength of these mice (Figure 7D) compared to the DEX treatment group. These results indicate that MON prevents a loss of mass and strength in mice with DEX-induced muscle atrophy.

## 4. Discussion

Muscle atrophy is a feature of sarcopenia that occurs often in elderly patients with chronic diseases such as type II diabetes mellitus (T2DM), chronic liver and kidney diseases, and chronic obstructive pulmonary disease (COPD) [14]. Treatment of muscle atrophy through nutritional supplements from plants is becoming more and more popular. Recently, effective herbs and their active compounds are known to help muscle health. For example, resveratrol supplementation is well-known to have many benefits in supporting muscle mass and function. It has been reported that resveratrol improves muscle mass by activating the ubiquitin–proteasome system, increases the mitochondrial content in diabetic muscle atrophy [15], and can favor muscle function by mediating endogenous antioxidant enzymes and apoptosis signals in hind limb suspension models [16]. Additionally, resveratrol inhibits the upregulation of Atrogin-1 and MuRF1 expression in DEX-stimulated L6 myotubes by the increase in SIRT1 expression [17] and attenuates TNF-α-induced muscle atrophy in C2C12 myotubes by regulation of protein synthesis metabolism [18]. However, in recent years, this compound has been known to have various functions such as altering metabolism, improving function, preventing cellular stress, and inhibiting protein catabolism in skeletal muscle [19]. However, it has not been found to explore specific mechanisms of protein synthesis anabolism in glucocorticoid-induced muscle atrophy as in our study. Quercetin is also a well-studied active compound in the improvement of muscle function. It has been reported that quercetin prevents muscle atrophy by regulation of the Bax/Bcl-2 expression in C2C12 cells [20], and increases the AKT phosphorylation with the regulation of the expression of myostatin and atrogenes in C2C12 myotubes and muscle tissues [21]. However, these studies do not suggest a specific mechanism, e.g., the AKT/mTOR/FOXO3a signaling pathway which plays an important role in the process of protein synthesis. In our study, it was firstly reported that the effect of MON on improving DEX-induced muscle atrophy appears by regulation of the AKT/mTOR/FOXO3a signaling pathway both in vivo and in vitro. We identified NON in Morindae Radix extract by HPLC analysis (Figure S2). In our aging society, many more studies to find more effective pharmacological strategies based on non-pharmacological methods will need to be carried out in the future. We will expect that MON can use as a useful natural source for the prevention and treatment of muscle atrophy.

In this study, the administration of MON to mice with DEX-induced muscle atrophy was an effective increase in muscle weights with increasing MyHC expression. MyHC is one of the major structural proteins of skeletal muscle and has the ability to resist gravity and maintain body movement [22]. The reduction in MyHC expression in muscle brought about by DEX treatment is considered to be an important pathological condition in atrophy [23,24]. Therefore, MON could help to prevent muscle atrophy by increasing the expression of MyHC in skeletal muscle.

Myostatin, known as a growth differentiation factor 8 (GDF-8), is a member of the transforming growth superfamily [25] and inhibits muscle growth as a negative regulator of muscle during embryonic development, myoblast differentiation, and muscle hypertrophy in adults [26]. Many studies have shown that DEX treatment induces an increase in Myostatin expression in C2C12 myotubes and reduces muscle weight through the up-regulation of Myostatin mRNA in C2C12 and L6 cells [27,28]. Therefore, Myostatin is considered to be an important factor in developing medicines for use in muscle atrophy therapy. In our study, Myostatin expression was increased by the administration of dexamethasone in the C2C12 myotubes and muscle tissues of mice; it was decreased in a dose-dependent manner by MON. This result indicates that MON can help to improve muscle atrophy.

Various studies have shown that protein degradation is mediated by the ubiquitin–proteasome pathways during the progression of muscle atrophy [29]. The hyper-activation of the ubiquitin–proteasome system increases the expression of Atrogin1 and MuRF1, which are key enzymes in the development of muscle atrophy that are triggered by the transcription of FOXO protein family members [30,31]. In this study, we investigated the regulatory effects of MON in muscle atrophy on inhibition of protein degradation markers, FOXO3a, Atrogin1, and MuRF1 and the activation of protein synthesis pathways, AKT, and mTOR. This suggests that MON could inhibit the ubiquitin/proteasome-dependent protein degradation pathway in patients with muscle atrophy.

The mTOR signaling pathway plays an important role in the process of protein synthesis. mTOR, a serine/threonine kinase, is involved in multiple cellular responses, such as cell growth and survival, and interacts with several proteins to form two distinct complexes, mTORC1, and mTORC2, respectively. In particular, mTORC1 signaling is known to trigger skeletal growth and metabolism by stimulation of protein synthesis [32,33]. In addition, it has been reported that mTORC1 inhibition increases protein degradation by the ubiquitin–proteasome pathway with the regulation of the muscle-specific E3 ubiquitin ligases such as Atrogin-1/MAFbx and MuRF1 in muscle homeostasis and atrophy [34,35]. However, additional molecular analysis that MON could affect the function of mTORC1 and mTORC2 in DEX-induced muscle atrophy is required for further studies.

PI3K/AKT signaling plays an important role in cellular physiology, including glucose metabolism in muscles [36]. In particular, AKT activation induces an increase in mTOR phosphorylation that positively regulates protein synthesis in muscles [37]. Therefore, the activation of the AKT/mTOR signaling pathway is considered to be able to prevent the development of muscle atrophy via promoting protein synthesis. In our study, MON increased the phosphorylation of mTOR and AKT in the C2C12 myotubes and muscle tissues of mice with dexamethasone-induced muscle atrophy. This indicates that MON can improve muscle atrophy through the enhancement of protein synthesis by activating the AKT/mTOR signaling pathways. Meanwhile, another important function of AKT is to regulate the expression of Atrogin1 and MuRF1 by regulating FOXO3a factors. AKT plays an important role in controlling mechanisms of protein synthesis and degradation in muscle mass [38]. In our results, MON increased AKT phosphorylation, leading to phosphorylation of FOXO3a, which inhibited Atrogin1 and MuRF1 expression both in muscle tissues and C2C12 myotubes. This indicates that the anti-atrophy effects of MON may be associated with stimulation of the AKT/mTOR/FOXO3a signaling pathways in muscle. In addition, Myostatin can dephosphorylate Akt by inactivation of FoxOs and activation of Smad2 [39].

Our result that the Akt phosphorylation increases by MON treatment appear to be due to the reduction of Myostatin expression. However, the molecular mechanism of MON on the myostatin regulation in muscle atrophy and the interaction of AKT/mTOR/FOXO3a signaling pathways require further studies. The anti-apoptotic effects of MON on IL-1β-induced chondrocytes have been reported. [10]. It may be that the anti-apoptosis effect is a direct influence on MON to improve muscle atrophy. In addition, MON’s anti-osteoporosis effects by an autophagy activation via the AKT/mTOR signaling pathway [11] and maybe help to alleviate muscle atrophy in myotubes and muscle tissues through this pathway. Dexamethasone, a synthetic glucocorticoid, induces muscle atrophy, which involves the induction of protein degradation and the suppression of protein synthesis in skeletal muscle by inhibiting the phosphorylation of the pHAS-I and p70S6K downstream regulatory sites of mTOR [40,41,42]. It has been shown previously that the long-term use of dexamethasone increases the expression of Myostatin and activates the dephosphorylation of the AKT pathway [43]. Therefore, by inhibiting AKT phosphorylation, the expression of active FOXO3a is also increased, which leads to the up-regulation of the expression of the muscle-specific E3 ubiquitin ligases, Atrogin1 and MuRF1 [44]. Muscle atrophy is associated with an imbalance in protein degradation/synthesis in muscle tissue. Due to the high similarity of the pathogenesis, the muscle atrophy induced by dexamethasone has been widely recognized by many researchers [45]. In this study, the expression of FOXO3a, Atrogin1, MuRF1 proteins in the protein degradation pathway, and AKT and mTOR in the protein synthesis pathway were detected. Additionally, the molecular mechanism exerted by MON on the downstream effector proteins of mTOR needs to be further identified.

## 5. Conclusions

In our study, it was firstly reported that MON improved dexamethasone-induced muscle atrophy by increasing muscle mass and strength in mice with dexamethasone-induced muscle atrophy. This effect was related to its mechanism of action, with an increase in MyHC expression and a decrease in Atrogin1, MuRF1, and Myostatin expression seen in the C2C12 myotubes and skeletal muscle tissues of mice that had undergone the activation of the AKT/mTOR/FOXO3a signaling pathway. This suggests that MON could be used as a natural drug for the prevention/treatment of muscle atrophy.

## Data Availability

Data are contained within the manuscript and Supplementary Materials.

## Supplementary Materials

The following supporting information can be downloaded at: https://www.mdpi.com/article/10.3390/nu14091859/s1, Figure S1: Effect of MON on cell viability in C2C12 cells; Figure S2: HPLC pattern of monotropein in Morindae Radix extract.
