# Supplementary material for: Monotropein Improves Dexamethasone-Induced Muscle Atrophy via the AKT/mTOR/FOXO3a Signaling Pathways
Source: Nutrients. 2022 Apr 29;14(9):1859. doi: 10.3390/nu14091859 (PMC9103778; doi:10.3390/nu14091859)
Supplement: Supplementary file 1 [file nutrients-14-01859-s001.zip › nutrients-1671600-supplementary.pdf]

# Suplement Information

## S1 Cell Viability Test

To check the change in cell viability induced by the MON treatment, an MTT assay was performed. Briefly, C2C12 myoblasts (6 × 103 cells in each well) were seeded in a 96-well plate with 100 ul of media and treated with different concentrations of MON (25 to 300 μM) with or without DEX (10 to 500 μM) for 24 h. Cell viability solution (0.5 mg/mL, DoGenBio Co., Seoul, Korea) was added to each well and the plate was stored at 37 °C for 2 h. The absorbance was measured at 450 nm using an ELISA microplate reader (Molecular Devices, Sunnyvale, CA, USA). Cell viability was expressed as a percentage relative to the normal or DEX-treated cells.

As a result, treatment with MON was shown to cause no significant difference in the cell viability up to 300 μM (Figure 1A). DEX treatment significantly decreased the viability at 100 ($p < 0.05$), 200 ($p < 0.05$), 300 ($p < 0.01$), and 500 μM ($p < 0.001$) in a concentration-dependent manner (Figure 1B). Based on the cell viability of DEX, 100 μM of DEX was used for the next study. MON treatment at levels of 50 ($p < 0.05$) and 100 μM ($p < 0.01$) significantly increased the cell viability in DEX-induced C2C12 cells (Figure 1C). These results indicated that MON can induce cell proliferation in DEX-induced C2C12 cells.

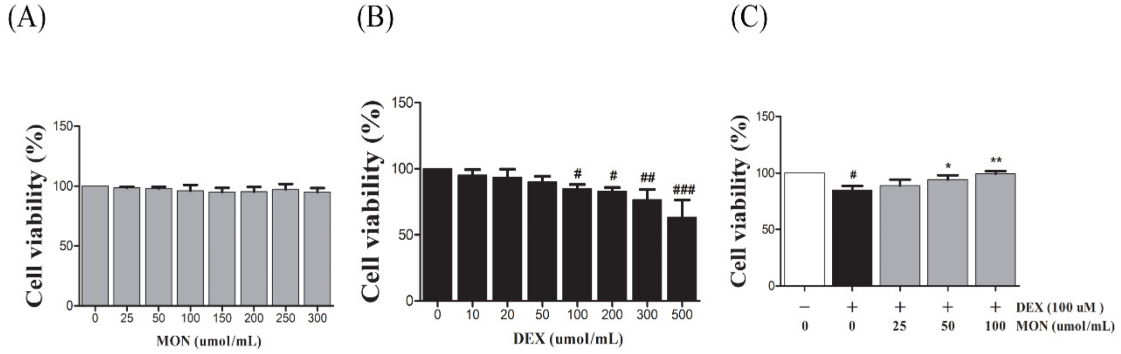


Figure S1: Effect of MON on cell viability in C2C12 cells. C2C12 cells were treated with MON (25–300 μM) (A) or DEX (10–500 μM) (B) and MON at 25, 50, and 100 μM with DEX for 24 h. (C) Cell viability was measured using an MTT assay. Each value is the mean ± SD of three independent experiments. # $p < 0.05$, ## $p < 0.01$, and ### $p < 0.001$ vs. Nor; * $p < 0.05$, ** $p < 0.01$, and *** $p < 0.001$ vs. DEX. Nor, normal group; DEX, dexamethasone-treated group; and MON, group administered monotropein.

## S2 High-Performance Liquid Chromatography (HPLC) Analysis

The content of monotropein in the Morindae Radix extract was detected using the Agilent 1260 infinity II quaternary system equipped with a G7129A vial sampler and a WR G7115 Adiode array detector (Agilent, Waldbronn, Germany). To prepare a work-ing solution, 0.198 g of Morindae Radix extract powder was dissolved in 4 mL of dis-tilled water and a concentration of 0.5 mg/mL of monotropein standard solution was made. Each solution was then filtered using a 0.45 μm membrane filter. Morindae Ra-dix extract and MON standard solution were analyzed using a C18 (150 mm × 4.6 mm, 5 microns) column (Agilent, Waldbronn, Germany). The mobile phase was methanol- 0.4% phosphate solution (10:90) (HPLC grade, Merck, Darmstadt, Germany), the flow rate was 1 ml/min, the detector wavelength was 231 nm, and the injection volumes were 10 μL and 5 μL.

To identify the content of MON in the Morindae Radix extract, we performed an HPLC analysis with a standard compound. As shown in Figure 7, the level of MON in the extract was 13.96 mg/g, as a main compound of Morindae Radix.

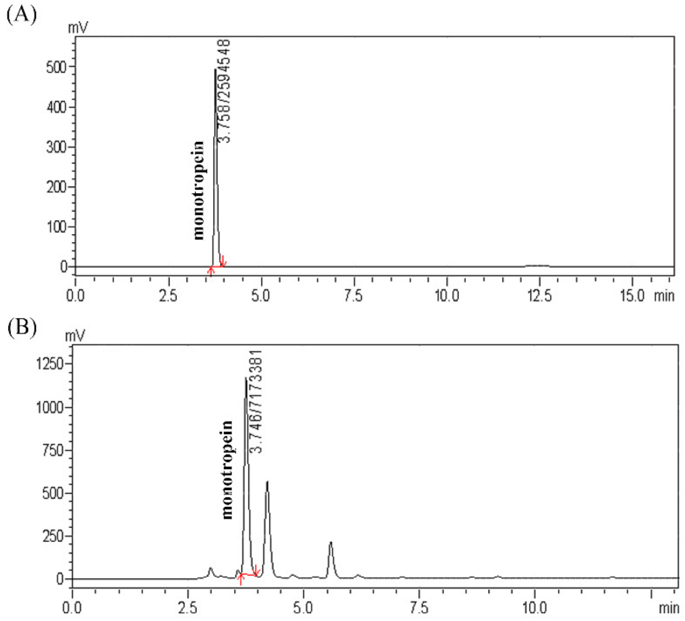


Figure S2:HPLC pattern of monotropein in Morindae Radix extract. Monotropein as a standard compound (A) and in Morindae Radix extract (B) at a retention time of 3.7 min.
